# Salvage of floral resources through re-absorption before flower abscission

**DOI:** 10.1038/s41598-020-72994-5

**Published:** 2020-09-29

**Authors:** Graham H. Pyke, Zong-Xin Ren, Judith Trunschke, Klaus Lunau, Hong Wang

**Affiliations:** 1 Key Laboratory for Plant Diversity and Biogeography of East Asia, Kunming Institute of Botany, Chinese Academy of Sciences, Kunming, 650201 People’s Republic of China; 2 Department of Biological Sciences, Macquarie University, Ryde, NSW 2019 Australia; 3 Institute of Sensory Ecology, Heinrich-Heine-University, Dusseldorf, Germany

**Keywords:** Ecology, Evolution, Plant sciences

## Abstract

Plants invest floral resources, including nectar and pigment, with likely consequent reproductive costs. We hypothesized that plants, whose flowers abscise with age, reabsorb nectar and pigment before abscission. This was tested with flowers of *Rhododendron decorum*, which has large, conspicuous white flowers that increasingly abscise corollas as flowers age. As this species is pollinated by bees, we also hypothesized that nectar concentration would be relatively high (i.e., > 30% wt/vol) and petals would contain UV-absorbing pigment. Floral nectar volume and concentration were sampled on successive days until abscission (up to ten days old, peak at five days) and for sub-sample of four-day-old flowers. Flowers just abscised were similarly sampled. Flower colours were measured using a modified camera, with recordings of spectral reflectance for abscised and open non-abscised flowers. Pigment content was summed values of red, green, blue channels of false color photos. As expected, flowers reabsorbed almost all nectar before abscission, separately reabsorbing nectar-sugar and nectar-water, and petals contained UV-absorbing pigment. However, flowers did not reabsorb pigment and nectar-concentration was < 30% wt/vol. That flowers reabsorb nectar, not pigment, remains unexplained, though possibly pigment reabsorption is uneconomical. Understanding floral resource reabsorption therefore requires determination of biochemical mechanisms, plus costs/benefits for individual plants.

## Introduction

Angiosperm plants invest resources in developing and maintaining flowers, thereby generally achieving sexual reproduction. Such resources may include the structure of the flower itself, with its stamens, style and perianth. Additional resources may include nectar, which acts as a food source for visiting nectar-feeding animals, some of which may be pollinators. They may also include floral pigments, which combine to produce the vast array of observed flower colour patterns^1–3^.

The investment by plants in these floral resources likely involves costs and tradeoffs to a plant. There may, for example, be a cost to a plant in producing floral nectar, as evidenced by a tradeoff between nectar production and other plant activities such as growth, seed production and production of extra-floral nectar^4–6^. The production of floral pigment should similarly entail a cost to a plant^7^.

It would therefore be expected that a plant would reabsorb floral nectar and floral pigment before flower petals abscise or separate from the plant (henceforth referred to simply as flower abscission), and then be able to reuse these resources. For many plant species, flowers may abscise and drop to the ground, especially as they age^8,9^. A number of studies have found that flowers may reabsorb secreted nectar as they senesce or deteriorate with age^10–14^, though none of these considers flower abscission. It has also been reported that senescing flowers may break down and reabsorb their organic components, including lipids, carbohydrates, proteins and nucleic acids, reusing the resulting chemical constituents in the ovaries or other plant parts^15–17^. It seems likely that flowers may similarly reabsorb and reuse the constituents of floral pigment, though this has not apparently been tested.

We therefore hypothesized that flowers exhibiting abscission as they age would reabsorb both floral nectar and floral pigment prior to flower abscission. We are not aware of any study that considers both these aspects of flower resource reabsorption.

**Figure 1:**
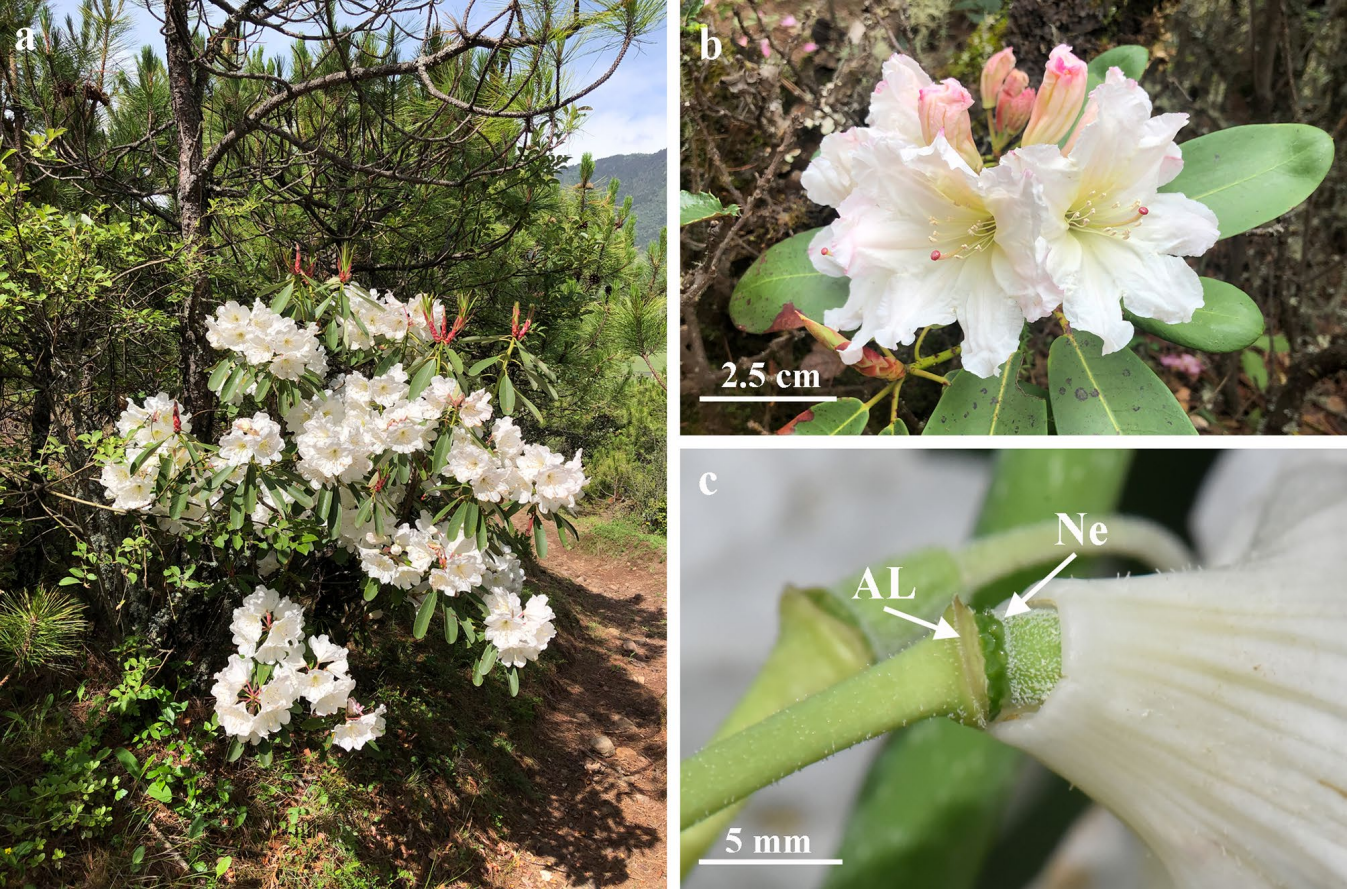
Plant, flowers, nectaries and abscission line of *Rhododendron decorum*. (**a**) A flowering individual plant; (**b**) large, conspicuous white flowers; (**c**) an abscised flower showing the location of nectaries (Ne) and abscission line (AL).

The plant species *Rhododendron decorum* provides an excellent opportunity to test this hypothesis. This plant species grows throughout southwest China and northern Myanmar at elevations from 1000 to 3600m^18^^,pers. obs.^, and has large, conspicuous white flowers that increasingly abscise and fall to the ground as they age (Fig. 1a,b,c; pers. obs.).

As the flowers of this plant species appear white from a human perspective (Fig. 1a,b) and are visited, and pollinated, primarily by bees^19^^,pers. obs.^, we expected that, at least prior to flower abscission, floral nectar sugar concentration would be relatively high (i.e., > 30% wt/vol) and petals would contain UV-absorbing pigment. Plants pollinated by bees generally secrete floral nectar with concentrations greater than 30% wt/vol^20^. Insect pollinated flowers, that appear white to the human eye, usually have UV-absorbing pigment in the petals, possibly because such animals may otherwise have difficulty differentiating between flowers and the visual background^21^.

Assuming that the production of floral nectar and floral pigment for *Rhododendron decorum* contribute to plant fitness through their roles in plant pollination, but entail fitness costs to a plant, we therefore hypothesized that, for this plant species:

- Floral nectar concentration would exceed 30% wt/vol prior to flower abscission, and would be significantly lower for flowers just before and just after flower abscission;
- Floral nectar volume, and floral nectar sugar weight, would both accumulate and hence increase with increasing flower age, but would then be significantly lower just after flower abscission;
- Flower petals would contain pigments, including UV-absorbing ones, but these would be reduced in post-abscission flowers relative to flowers pre-abscission.

## Methods

### General

This study was carried out in the Lijiang Forest Ecosystem Research Station, Yunnan Province, China during the period 13 July to 3 August 2019. This field station, which is operated by the Kunming Institute of Botany, Chinese Academy of Sciences, is located to the north of Lijiang on Yulong Snow Mountain at an elevation of 3200 m. Canopy vegetation is dominated by *Pinus yunnanensis* and *Quercus variabilis*, and *Rhododendron decorum* is a highly conspicuous understory shrub species, when in flower. Flowers occur in inflorescences with each plant typically having many inflorescences.

We chose, numbered and bagged one inflorescence on each of 25 plants of *Rhododendron decorum*, followed the state of individual flowers, and sampled nectar according to a protocol explained below. We selected plants, as encountered, that were flowering and within about 10 m of our walking path, which was along a road and foot track near the field station. We selected inflorescences, one per plant, with at least five unopened buds, and marked five of these buds with small lengths of differently coloured plastic drinking straws^22^. The different colours enabled us to distinguish flowers during nectar sampling and subsequent measurements of flower colour. All marked flowers were then checked daily to record flower state as bud, beginning to open, open-non-abscised, and open-abscised. Flowers were considered buds if there was no sign of petals unfolding, beginning to open if petals had begun to unfold, and open if petals had unfolded completely. Abscised flowers were clearly indicated by separation between the base of the petals and the rest of a flower, which was along a distinct abscission line (Fig. 1c). Inflorescences were bagged, using green mesh organza bags, to prevent any flower visitation and nectar removal. This species is self-incompatible^19^, so no pollination occurred.

We carried out two experiments, involving a total of 25 plants. One (Experiment A) involved 10 plants (numbered A-1 to A-10) and was carried out between 13 and 23 July 2019. Experiment B involved 15 plants (numbered B-1 to B-15) and was carried out between 24 July and 3 August 2019. Experiment B was carried out to increase sample sizes for flowers of all ages, and to provide information for relatively young flowers that was not provided by Experiment A (explained further below). Plants were numbered as encountered.

### Collection of inflorescences

Inflorescences from Experiment A and Experiment B were collected for sampling of nectar according to the following protocol.

For all inflorescences in Experiment A (i.e., 10 inflorescences) and all in Experiment B, except numbers B-3, 6, 9, 12 & 15 (i.e., 10 inflorescences), each inflorescence was removed from its plant on the first day that abscission of any marked flower was observed. If any marked flower was observed to have abscised, its inflorescence was removed from its plant by breaking its subtending stem and taken to a nearby sheltered ‘nectar sampling station’ where nectar measurements were made. This occurred for flowers between 3 and 9 days of age, counting the first day that a flower was either open or beginning to open as age 1. In a small number of cases, flower abscission occurred when marked flowers were gently touched just prior to nectar sampling. Such flowers were also considered to have abscised.

In addition, five inflorescences from Experiment B (i.e., numbers B-3, 6, 9, 12 & 15) were similarly collected when they were four days old, regardless of whether any flowers had abscised. This provided nectar measurements for relatively young flowers (i.e., ages 1 to 4 days).

### Nectar sampling

For collected inflorescences, almost all the marked flowers were open, and we sampled accumulated nectar in each marked and open flower as follows. Nectar was removed using micro-capillary tubes (Hirschmann microcapillary pipettes; 5 µl in Experiment A; 10 µl in Experiment B; both 32 mm long), with volume measured on the basis of nectar length along tube and subsequently converted to µl. When about 0.5 µl of nectar was obtained, this was expelled to a hand-held refractometer (i.e., Bellingham & Stanley, 0 to 50% brix, adjusted for small volumes) for measurement of sugar concentration as % wt/wt sucrose equivalents. These measurements were adjusted for ambient temperature (see Supplementary Information) using a formula developed from information supplied by the manufacturers of the refractometers we use^23^ and converted to wt/vol using the following formula^24^: Y = 0.00226 + 0.00937X + 0.0000585X^2^ where Y is sugar mass per unit volume (mg/µl) and X is % concentration wt/wt. The amount of sugar for a flower (in mg) was then calculated by multiplying nectar volume (µl) by sugar mass per unit volume (mg/ µl).

Nectar was sampled, for both abscised and non-abscised flowers, from where it accumulates after secretion (Fig. 1c). Nectar was sampled for non-abscised flowers from the base of the corolla between the ring of about 10–15 nectaries, around the base of the ovary, and adjacent flower petals. For flowers that had abscised, nectar was separately sampled from both the ring of nectaries and the inside lowest 5 mm of the flower petals, where some nectar becomes attached.

Some flowers were judged to have been affected by rain and their nectar concentration measurements were excluded from analyses. There were periods of rain during our study and occasionally nectar concentration readings of lower than 1.5% wt/wt were obtained (n = 6), and the nectar assumed to have been diluted by rainwater. These records were excluded from analyses. Fortunately, most flowers pointed downwards and were thus not affected by rain.

### Flower colour and pigment

Flower colours were measured by means of a modified Panasonic GH-1 camera. The low-pass filter of the camera had been removed in order to increase the sensitivity for ultraviolet light. The camera body was combined to an Ultra-Achromatic-Takumar 1:4.5/85 lens made of fused quartz that transmits UV light. Since the modified camera is sensitive to ultraviolet and infrared light, a UV-/IR-Cut filter transmitting light between 400 nm and 700 only nm was used to capture a normal reference picture. In addition, a UV-picture was captured from the identical position using a Baader UV-filter that transmits near ultraviolet light only. A white Teflon disc reflecting equal amounts of light in a range of wavelength from 300 to 700 nm was used for manual white balance before taking pictures. Using Image J both pictures were split into the RGB color channels, and then a false color photo was merged using the green channel of the color picture as red, the blue channel of the color picture as green, and the blue channel of the UV picture as blue (see Supplementary Information). For more details see article by Verhoeven et al.^25^. Using IrfanView image’s histogram a uniform non-decomposed area (number of pixels > 10,000) of the adaxial corolla on the false color picture was selected. The average intensity for the red, green and blue channel of the false color photos with values between 0 and 255 was used for color evaluation. Abscised and non-abscised flowers were photographed together enabling direct comparison of the colours of the flowers.

Pigment content was deduced from the sum of the values of the red, green and blue channel of the false color photos. Since abscised and non-abscised flowers both appear white to the human eye, the possible change in the content of a UV-absorbing pigment was checked by comparing the value for the blue channel in relation to the sum for the values of the green and red channels.

Recordings of the spectral reflectance were done with an abscised and an open, non-abscised flower from each of five inflorescences. Reflectance measurements were performed with an USB2000 + spectrophotometer (Ocean Optics) and illumination was provided by a DH-2000-BAL light-source (Ocean Optics), both connected via a coaxial fibre cable. All measurements were taken in an angle of 90° to the measuring spot with a pellet of barium sulphate used as white standard and a black piece of plastic used as black standard.

### Analyses

We used the General Linear Model approach to determine relationships for all flowers between nectar attributes (i.e., volume—µl, concentration—wt/vol, sugar weight—µg) as dependent variables and flower age, whether abscised, experiment (i.e., A vs. B), and Plant ID as independent variables. We also treated Plant ID as an independent categorical variable, but nested within experiment.

We used ANOVA to evaluate relationships between reflectance intensity and whether flower abscised, across different false colours, with Kolmogorov–Smirnov test for normality and Tukey post-hoc comparisons between means. We took log intensity as the dependent variable in order to meet the normality assumption.

We compared spectral reflectance for abscised and open, non-abscised flowers on the basis of the average reflectance across all wavelengths. Here we assumed that the 1140 reflectance values for each flower could be combined into a single average measure and that this average measure adequately represented each flower. We compared the two groups of flowers with a Kolmogorov–Smirnov Two Sample Test.

All analyses were carried out using the software SYSTAT^26^.

## Results

### Floral abscission

**Figure 2:**
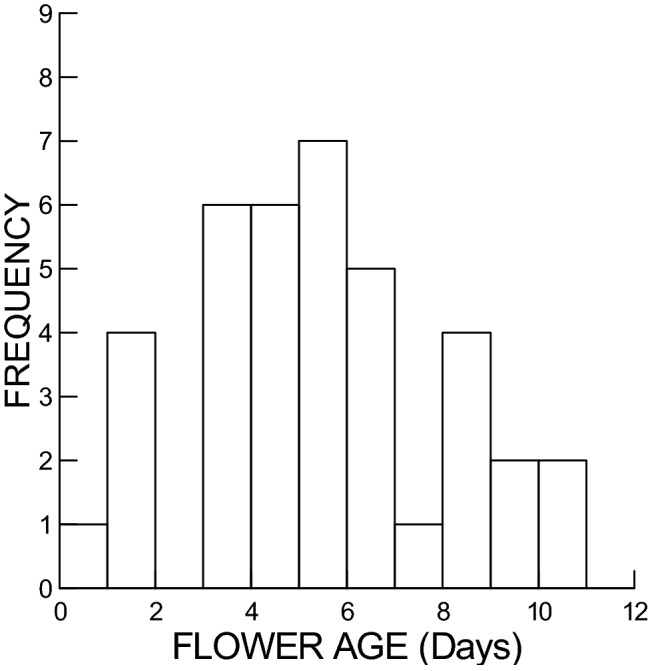
Frequency distribution of flower ages at time of abscission (Bud is Age = 0; day when flower is first open or beginning to open is Age = 1 day).

Floral abscission was recorded for 38 flowers and occurred for flowers that ranged in age from 0 (i.e., still bud) to 10 days old, with a peak at about age 5 days (Fig. 2). Consequently, we had nectar sampling measurements across this range of flower ages, for both flowers that are open and not yet abscised and flowers that have abscised. Other flowers either had not abscised, or were collected and sampled when four days old regardless of whether or not they had abscised.

### Nectar sampling

**Figure 3:**
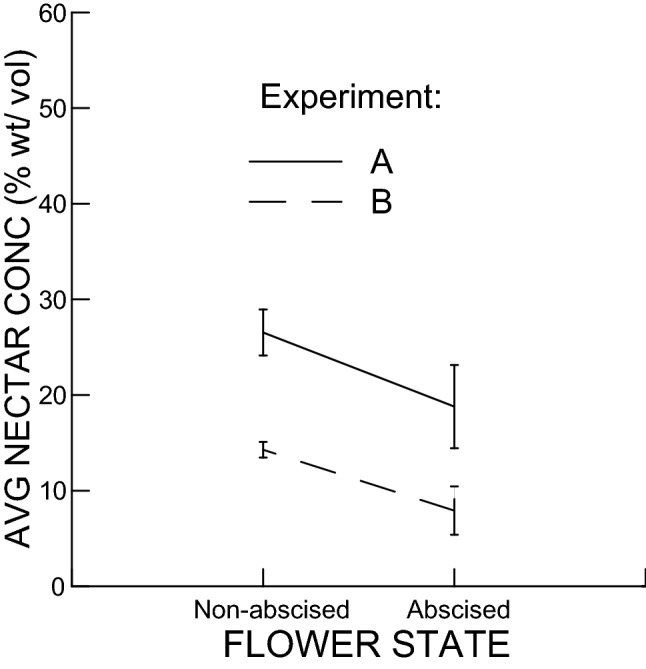
Average nectar sugar concentration vs. Flower state (i.e., Open, non-abscised vs. Abscised), separately for experiments A & B. Sugar concentration was significantly lower for abscised flowers and for experiment B.

Average nectar sugar concentration per flower was lower for abscised than open, non-abscised flowers, and lower for experiment B than for experiment A (Fig. 3). Both these factors were significant (flower state: F-Ratio = 7.69, df = 1&72, *P* = 0.007; experiment: F-Ratio = 34.0, df = 1&72; *P* < 0.005), while flower age was not significant (F-Ratio = 0.63, df = 1&72, *P* = 0.43). In this case Plant ID was also significant (F-Ratio = 10.78, df = 23&72, *P* < 0.005).

**Figure 4:**
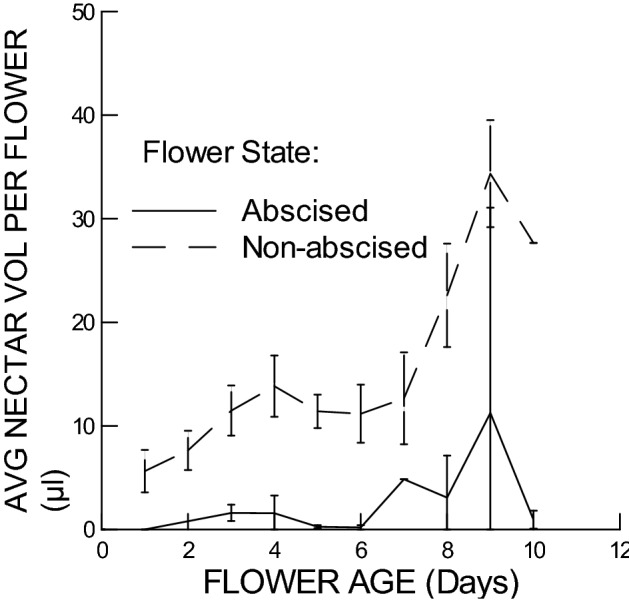
Average nectar volume per flower (µl) vs. Flower Age, separately for open, non-abscised flowers and abscised flowers. In both cases, average nectar volume increased significantly with flower age, but abscised flowers had significantly lower nectar volumes than non-abscised flowers.

Average nectar volume per flower increased with flower age for both open non-abscised flowers and abscised flowers, but was lower for abscised than non-abscised flowers (Fig. 4). Both these factors were significant (Age: F-Ratio = 24.8, df = 1&92, *P* < 0.005; Flower State: F-Ratio = 50.5; df = 1&92, *P* < 0.005), while experiment was not significant (F-Ratio = 1.74, df = 1&92, *P* = 0.19). Plant ID was again significant (F-Ratio = 1.98, df = 24&92, *P* = 0.01).

**Figure 5:**
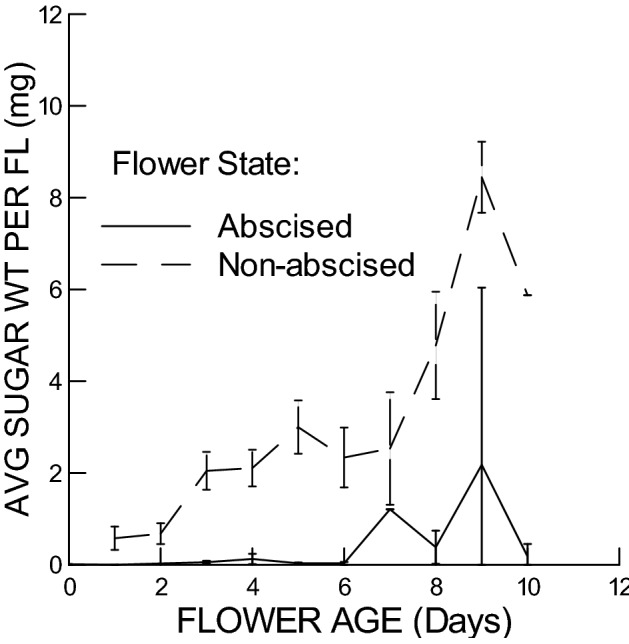
Average nectar sugar weight per flower (mg) vs. Flower Age, separately for open, non-abscised flowers and abscised flowers. In both cases, average nectar sugar weight increased significantly with flower age, but abscised flowers had significantly lower nectar sugar weights than non-abscised flowers.

Average sugar weight per flower similarly increased with flower age for both open non-abscised flowers and abscised flowers, but was lower for abscised than non-abscised flowers (Fig. 5). Both these factors were significant (Age: F-Ratio = 35.4, df = 1&116, *P* < 0.005; Flower State: F-Ratio = 43.0, df = 1&116, *P* < 0.005), while Experiment was not significant (F-Ratio = 2.56, df = 1&116; *P* = 0.11). Plant ID was again significant (F-Ratio = 2.11, df = 24&116, *P* = 0.006).

For open non-abscised flowers, nectar volume accumulated at an average rate of 2.32 µl per flower per day (s.e.m. = 0.47 µl; n = 87; GLM; Fig. 4), with an average sugar concentration that ranged from 14.3 to 26.5% wt/vol, depending on the experiment (Expt A: mean = 26.5% wt/vol, s.e.m. = 2.4%, n = 37; Expt B: mean = 14.3% wt/vol, s.e.m. = 0.80, n = 47; Fig. 3).

### Colour and pigment

**Figure 6:**
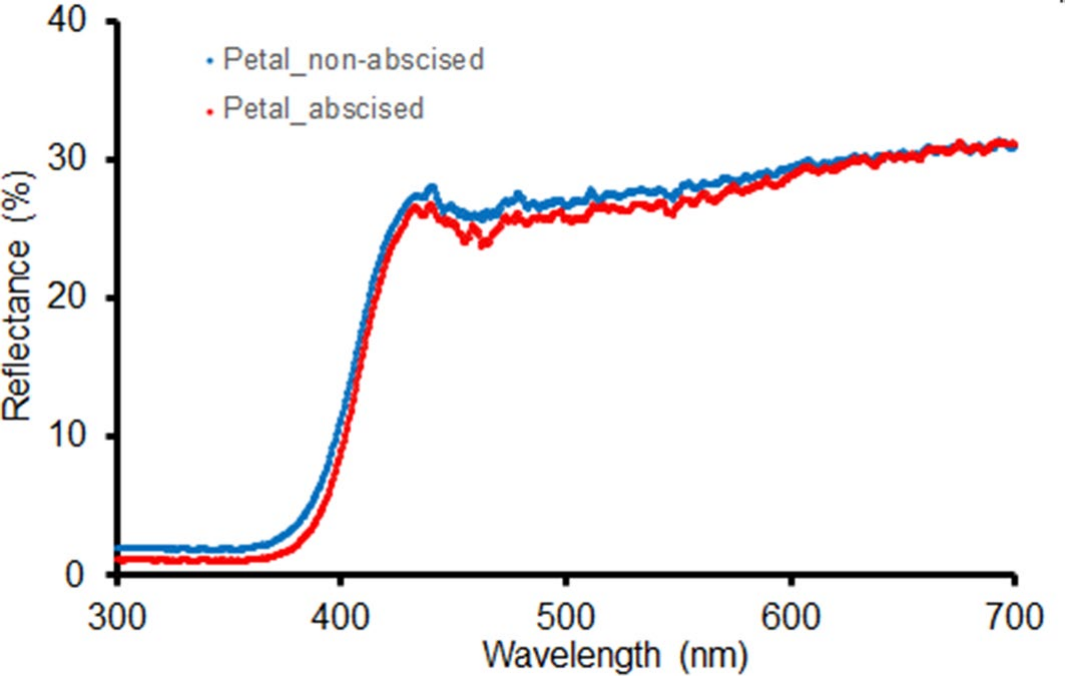
Mean spectral reflectance of the adaxial side of the petals of non-abscised and of abscised *Rhododendron decorum* flowers average from 5 recordings each.

The spectral reflectance curves of white parts of the adaxial side of the corolla were very similar for abscised and non-abscised flowers and both show strong absorption in the UV-range of wavelengths (Fig. 6). The difference in average reflectance between the two groups of flowers was not significant (*P* = 0.8, Kolmogorov–Smirnov Two-sample Test).

**Figure 7:**
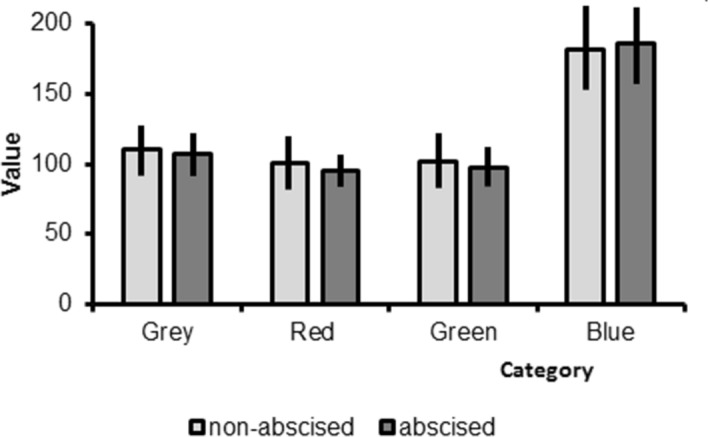
Values of intensity for all channels, the red, green, and blue channel representing the green, blue, and ultraviolet range of wavelengths for non-abscised and abscised *Rhododendron decorum* flowers. Reflectance intensity was significantly greater for the blue false colour channel than for the grey colour channel and the other false colour channels, but there was no significant difference between abscised open and non-abscised flowers.

Reflectance intensity was significantly greater for the blue false colour channel than for the grey colour channel and the other false colour channels, but there was no significant difference between abscised open and non-abscised flowers (Fig. 7; False Colour: F-ratio = 106.3, df = 3, *P* < 0.001; Abscised vs. non-abscised: F-ratio = 2.22, df = 1, *P* = 0.14; Comparisons between blue & other colours: All *P* < 0.001).

False colour photographs of flowers of *Rhododendron decorum* also indicate little difference between abscised and non-abscised corollas (see Supplementary Information).

## Discussion

### Pollination syndrome

Our observations in terms of floral nectar and floral pigment differed only slightly from what we expected based on bee pollination of our subject plant. The observed average nectar sugar concentrations, which ranged from about 14–27% wt/vol, were lower than expected^20^. However, the presence of UV-absorbing pigment was as expected, since the flowers are white to human eyes, but visited and pollinated by bees^21^. Further information in relation to pollination syndromes involving plants and their pollinators should be provided by our ongoing research in our study area on Yulong Snow Mountain for nectar and flower colour across many plant species, including other *Rhododendron* species.

### Floral nectar

As expected, flowers reabsorbed almost all of their nectar sugar before they abscised. Nectar sugar weight accumulated in open non-abscised flowers at an average rate of 0.519 µg per flower per day (GLM, s.e.m. = 0.055, n = 48), compared with 0.013 µg per flower per day for abscised flowers of the same ages (GLM, s.e.m. = 0.005, n = 26; excl. constant which is not significantly different from zero). Thus, the flowers must have reabsorbed about 97% of their nectar sugar before abscission (i.e., 0.506/ 0.519). Similarly high levels of nectar reabsorption as flowers senesce have been reported for some other plant species e.g.,^5,11,13^ and implied to be a common phenomenon^27^.

Before flower abscission, flowers must have reabsorbed nectar water as well as the nectar sugar, but with a preference for the nectar sugar component. Both nectar volume and nectar sugar were much reduced in abscised flowers compared with open, non-abscised flowers (Figs. 4 and 5), indicating that both nectar water and nectar sugar were reabsorbed, possibly in combination. However, nectar sugar concentration was also reduced in abscised flowers relative to open, non-abscised flowers (Fig. 3), at the same time that volume decreased, which indicates that hygroscopic dilution of nectar did not occur, and the flowers must have separately and preferentially reabsorbed the nectar sugar. Separate reabsorption of nectar water and nectar sugar has similarly been found in other studies^11,28–30^.

The cytological and molecular mechanisms whereby flowers may differentially reabsorb nectar water and nectar sugar are presently unknown, though it is clear that a variety of potential physical and biochemical pathways exist^29^. Just as nectar may be secreted via modified stomata or nectary epidermal cells, the processes can be reversed with consequent reabsorption of nectar water, sugars and other substances^29,31^. Nectar may be secreted and reabsorbed, perhaps somewhat passively, through direct interchange between nectar and phloem^32^, or through cells with appropriate biochemical machinery^31^.

### Floral pigment

Contrary to expectation, there was no apparent absorption of floral pigment prior to flower abscission. Spectral reflectance indicated the presence of UV-absorbing pigment, for both open, non-abscised flowers and flowers that had abscised (Fig. 6). Spectral reflectance varied little from blue to red (i.e., across wavelengths between about 430 and 700 nm), with no significant difference between abscised and non-abscised flowers (Fig. 6). The intensity of reflectance was higher for blue than the other colours, but there was no significant difference between abscised and non-abscised flowers, indicating an absence of pigment differences between the two kinds of flower and hence absence of pigment resorption prior to flower abscission (Fig. 7).

A possible explanation for this absence of pigment reabsorption is that it is uneconomical for the plants. This could arise if the gains from pigment reabsorption are relatively small, as could occur if pigment concentrations are low or key constituents such as N are not present. It could also arise if the costs of chemical breakdown are relatively high or if pigment reabsorption is otherwise metabolically difficult. In general, reabsorption of floral resources is only expected if there is a consequent net benefit^14,33^.

### Future research

To comprehensively understand the nature and extent of floral resource reabsorption, it will be necessary to consider more plant species and to better determine the biochemical mechanisms involved, as well as the costs and benefits to individual plants. Plant species with different floral nectar and pigmentation may, for example, vary in the extent to which they reabsorb these floral resources. In the case of pigment reabsorption, biochemical mechanisms may depend on the particular pigments involved and their molecular structures, the presence of necessary enzymes and other chemicals to break down these pigments, and the existence of pathways whereby breakdown products can be transported to sites where they can be recycled. In the case of sugar reabsorption, though relatively simple molecules are involved, biochemical mechanisms may nonetheless vary with the kinds of sugar molecule involved and transport pathways. Such biochemical mechanisms, including breakdown and transport, may require energy and other ingredients, and may thus incur costs. Benefits will depend on where, when and how any breakdown products can be utilized to enhance the biological fitness of individual plants, such as may occur if resources are transported to the ovary where they promote plant reproduction. All of this warrants further research.

### Conclusions

We have demonstrated that flowers of *Rhododendron decorum* reabsorb, as expected, almost all floral nectar remaining prior to flower abscission, but they do not reabsorb floral pigment. However, it remains unclear why or how they behave this way.

## Supplementary information

Supplementary Information.

## Data Availability

Data will be lodged in an appropriate repository, such as Dryad.
