# Supplementary material for: Salvage of floral resources through re-absorption before flower abscission
Source: Sci Rep. 2020 Sep 29;10:15960. doi: 10.1038/s41598-020-72994-5 (PMC7524801; doi:10.1038/s41598-020-72994-5)
Supplement: Supplementary file 1 — Supplementary Information. [file 41598_2020_72994_MOESM1_ESM.docx]

Salvage of floral resources through re-absorption before flower abscission

Graham H. Pyke^1,2,*^, Zong-Xin Ren^1,*^, Judith Trunschke^1^, Klaus Lunau^3,1^ & Hong Wang^1^

1. Key Laboratory for Plant Diversity and Biogeography of East Asia, Kunming Institute of Botany, Chinese Academy of Sciences, CN-650201 Kunming, PR China
2. Dept of Biological Sciences, Macquarie University, Ryde, NSW 2019, Australia
3. Institute of Sensory Ecology, Heinrich-Heine-University, Dusseldorf, Germany

Supplementary information

*Temperature adjustment for refractometer readings*

We adjusted refractometer readings for nectar concentration (wt/wt) with a formula derived from a temperature compensation table provided by the manufacturers of the refractometers we used ^1^. This table provides required adjustments for recorded sugar concentration that depend on the observed concentration reading and ambient temperature at the time. Plotting mean adjustments against temperature (T) and concentration (C) suggested slightly curvilinear relationships. We therefore used the General Linear Model, Least Squares estimation, to determine the unknown constants in the following equation: A_0_ + A_1_ x T + A_2_ x C + A_3_ x T^2^ + A_4_ x C^2^. We found that A_0_ = -1.307 (s.e. = 0.0166), A_1_ = 55.08 x 10^-3^ (s.e. = 1.25 x 10^-3^), A_2_ = 1.934 x 10^-3^ (s.e. = 0.166 x 10^-3^), A_3_ = 0.475 x 10^-3^ (s.e. = 0.023 x 10^-3^), and A_4_ = -0.027 x 10^-3^ (s.e. = 0.002 x 10^-3^), with R^2^ =0.998. We consequently used this equation to adjust all sugar concentration readings with the assumption that ambient temperature was the average of the start and end temperatures observed during our nectar sampling periods which lasted 1-2 hours. In the few cases where this resulted in negative concentrations after adjustment, we took the adjusted concentration to be zero.

*Reference*

1 Bellingham & Stanley. (Bellingham & Stanley, Tunbridge Wells, Kent, UK, 2013).

*Bee colour vision and false colour photography*

The sensitivity of the colour vision system in bees is shifted towards shorter wavelength as compared to humans. Honeybees and humans possess three types of photoreceptors. The maximal sensitivity of the photoreceptors in honeybees is in the ultraviolet, blue, and green, those of humans in the blue, green, and red range of wavelengths. False colour photography of flowers in bee view is based on the range of wavelength visible to bees. A false colour picture is merged from a UV-photo and a colour photo that have been split into the red, green, and blue channels. For the merged false colour picture, the UV channel of the UV-photo has been used as the new blue channel, the blue channel of the colour photo has been used as the new green channel, and the green channel of the colour photo has been used as the new red channel. The green and red channels of the UV-photo, and the red channel of the colour photo have been discarded.

False colour photographs of flowers of *Rhododendron decorum* indicate little difference between abscised and non-abscised corollas (Fig. S1).


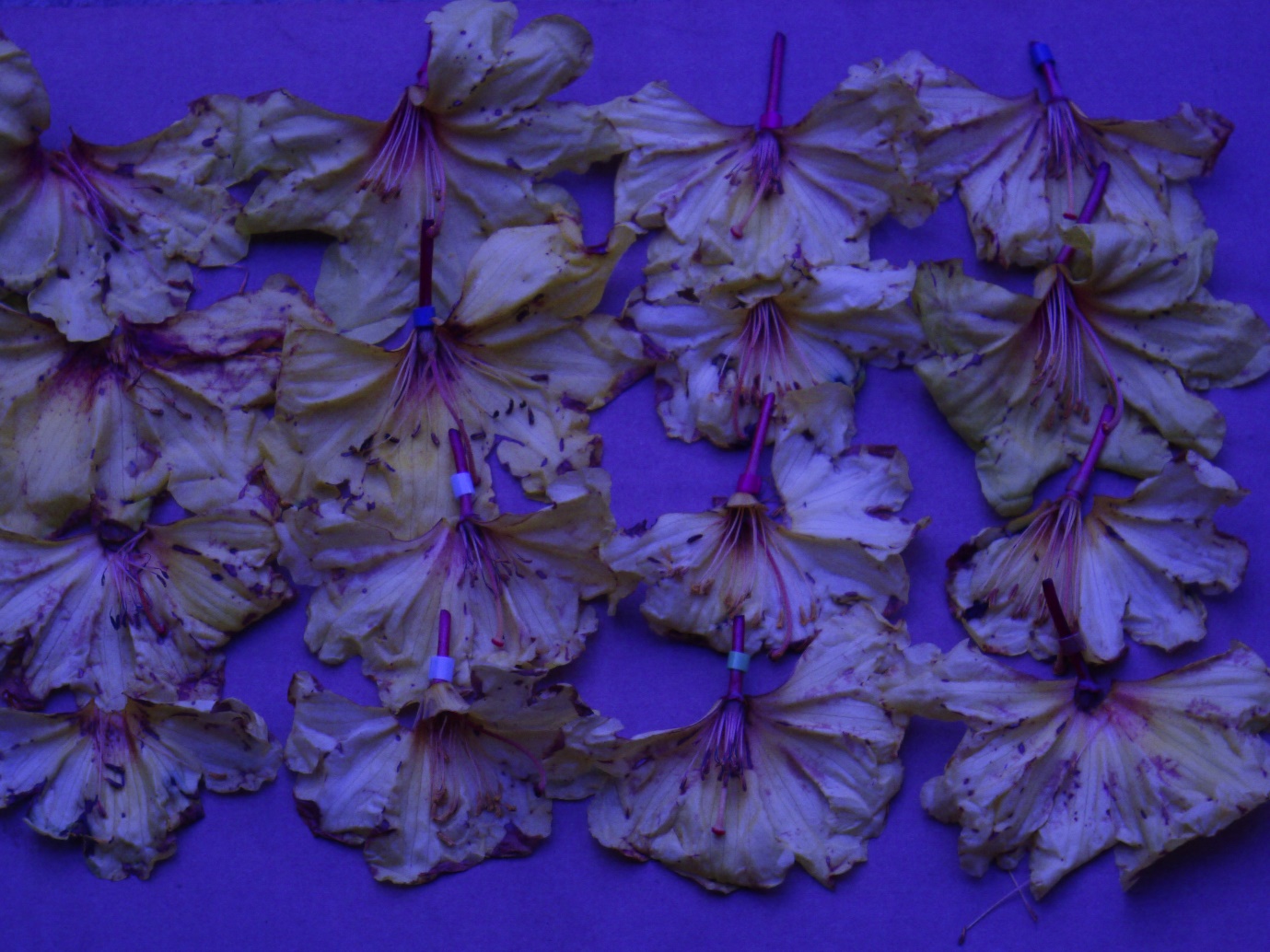


Fig. S1 False colour picture of adaxial side of 4 abscised (left) and 12 non-abscised corollas of *Rhododendron decorum*.
